# Sexual Dysfunction Is Common in Reproductive-Age Women with Systemic Sclerosis

**DOI:** 10.3390/life15091441

**Published:** 2025-09-14

**Authors:** Lingling Salang, Pranom Buppasiri, Arporn Jutiviboonsuk, Chingching Foocharoen

**Affiliations:** 1 Department of Obstetrics-Gynecology, Faculty of Medicine, Khon Kaen University, Khon Kaen 40002, Thailand; slingling@kku.ac.th (L.S.);; 2 Division of Rheumatology, Department of Medicine, Faculty of Medicine, Khon Kaen University, Khon Kaen 40002, Thailand

**Keywords:** systemic sclerosis, scleroderma and related disorders, reproductive age, sexual impairment, sexual dysfunction, sexual function

## Abstract

**Background:** Female sexual dysfunction (FSD) is an underrecognized issue in women with systemic sclerosis (SSc), influenced by physical and psychological factors. Data on FSD in reproductive-age SSc patients, especially those with diffuse cutaneous SSc (dcSSc), remain limited. **Objectives:** This study aimed to determine the prevalence of FSD and identify its associated factors among reproductive-age women with SSc. **Methods:** A cross-sectional study (May 2019–March 2020) included sexually active women with SSc aged 18–45. Patients with surgical amenorrhea, prior radiation, hormonal contraceptive use within 12 weeks, or pregnancy were excluded. Sexual function was assessed using the Female Sexual Function Index (FSFI). **Results:** Among 27 women of reproductive age, 66.7% had the dcSSc subset. The mean age was 39.4 ± 5.2 years (range: 22–45 years), with a mean disease duration of 9.9 ± 7.9 years. FSD was identified in 51.9% of patients (95%CI: 31.9–71.3), with a higher prevalence in the dcSSc subset (71.4%) compared to limited cutaneous SSc (28.6%). Patients with FSD were more likely to be older at disease onset, exhibit telangiectasia, and have longer exposure to cyclophosphamide (CYC), although these findings were not statistically significant. Women with FSD showed significantly lower FSFI scores in arousal, lubrication, orgasm, sexual satisfaction, and total sexual function (*p* < 0.01 for all). **Conclusions:** FSD is highly prevalent among SSc women of reproductive age, particularly in those with dcSSc. Disease severity, older age at onset, and prolonged CYC treatment may contribute to the risk of FSD. Early recognition and management of sexual health issues are essential in this patient population.

## 1. Introduction

Systemic sclerosis (SSc) is a rare multisystem connective tissue disease characterized by inflammation, autoimmunity, vasculopathy, and fibrosis of skin and internal organs [1]. The disease is classified into two subsets based on the extent of skin involvement: limited cutaneous systemic sclerosis (lcSSc) and diffuse cutaneous systemic sclerosis (dcSSc) [2]. In lcSSc, skin involvement is limited to hands, face, feet, and forearms, and pulmonary arterial hypertension (PAH) frequently occurs. In contrast, dcSSc is a rapidly progressive skin disease that affects larger areas and is associated with internal organ involvement, including the kidneys, lungs, and heart. SSc predominantly affects women, with a female to male ratio ranging from 3:1 to 14:1 and the peak age at onset was reported around 45–69 years [3,4]. However, SSc is not limited to middle age and older populations; a substantial number of cases develop during reproductive years [5,6,7]. This demographic pattern creates a unique intersection of chronic disease management and reproductive health concerns.

Despite the potential for pregnancy in women with SSc, the prevalence of pregnancy among these patients remains notably low [8]. This reduced fertility pattern likely stems from multiple factors. While the late-onset nature of SSc partially explains this trend, other contributing elements deserve careful consideration. Many women diagnosed with SSc may consciously decide against childbearing due to concerns about disease progression, medication risks, or overall health stability. Additionally, biological factors may play significant roles, including premature ovarian insufficiency, diminished ovarian reserve, and perhaps sexual dysfunction.

Female sexual dysfunction (FSD) is common in women with chronic diseases [9] and has also been reported in SSc [10]. FSD in SSc may be induced by physiology and psychologic changes, including skin thickening, vaginal tightness and dryness, depression, and fatigue [11,12]. A decrease in the frequency of sexual activity in female SSc patients has been reported to be related to vaginal dryness, painful intercourse, and reduced intensity of orgasm [13,14].

Most Thai SSc patients (70%) have dcSSc, in contrast to most Caucasians and some Asians (17–37%) in whom lcSSc is more common [15,16,17]. We hypothesized that the prevalence of FSD might be higher in patients with dcSSc than in those with lcSSc, as dcSSc is the more common SSc subset among Thais and is associated with disease severity, particularly internal organ involvement. Therefore, our objectives were to evaluate the prevalence and associated factors of FSD among Thai women with SSc.

## 2. Materials and Methods

### 2.1. Patient Population and Inclusion Criteria

A cross-sectional study was conducted among adult female SSc patients aged 18–45 years who attended the Scleroderma Clinic, Khon Kaen University, Khon Kaen, Thailand, between October 2019 and March 2020. All included patients had a diagnosis of SSc based on the classification criteria for systemic sclerosis by the ACR/EULAR 2013 [18] and were sexually active. We excluded patients with surgical amenorrhea, a history of previous radiation, use of hormonal contraception within the past 12 weeks, and those who were pregnant. Sexual function was assessed using the Thai version of the validated Female Sexual Function Index (FSFI) questionnaire [19]. Demographic data, comorbid diseases, obstetrics and gynecologic history, clinical characteristics of SSc, and concomitant medication were also collected.

### 2.2. Questionnaire

The FSFI questionnaire is a questionnaire specifically designed and validated to evaluate female sexual function. It consists of six domains and 19 items, including sexual desire (2 questions), sexual arousal (4 questions), lubrication (4 questions), orgasm (3 questions), sexual satisfaction (3 questions), and pain during sexual intercourse (3 questions). Each item is scored on a scale from 0 to 5, except for items 1, 2, 15, and 16, which are scored from 1 to 5.

To calculate the domain scores, the sum of the scores within each domain is multiplied by a specific domain factor. The total score is obtained by summing the six domain scores, with a maximum possible score of 36. Higher scores indicate better function in each domain. A total score of less than 26.55 is indicative of FSD [20].

### 2.3. Operational Definitions

SSc was classified as the limited or diffuse subset as per LeRoy et al. [2]. Onset of disease is considered the date of first non-Raynaud symptoms. Digital ulcer is defined as painful denuded areas with well-demarcated borders located on the volar aspect of the fingers [21]. Hand deformity is characterized by flexion contracture of the finger joints or claw deformities [22]. Interstitial lung disease (ILD) is diagnosed when interstitial fibrosis is detected by high-resolution computed tomography. PAH is confirmed when the mean pulmonary arterial pressure exceeds 20 mmHg at rest, with a pulmonary artery wedge pressure of ≤15 mmHg and a pulmonary vascular resistance of ≥3 Wood units, as assessed by right heart catheterization [23]. Left ventricular systolic dysfunction is identified by a conventional echocardiographic ejection fraction of less than 50%, regardless of the presence or absence of cardiac symptoms [24]. Diastolic dysfunction was considered present when the tissue Doppler longitudinal velocity of the septal mitral annulus was less than 8 cm/s [25]. Esophageal involvement is defined by the presence of any esophageal symptoms associated with SSc (i.e., esophageal dysphagia, heartburn, or reflux symptoms). Stomach involvement is identified by symptoms such as early satiety or vomiting [26]. Intestinal involvement is determined by the presence of symptoms including bloating, diarrhea, constipation, malabsorption, and/or signs of ileus or pseudo-intestinal obstruction. Renal crisis is identified by one or more of the following: (a) a rapid and progressive rise in serum creatinine, (b) the abrupt onset of hypertension, and/or (c) microangiopathic hemolytic anemia. Menopause is characterized by the absence of menstruation for 12 consecutive months [27]. Early menopause is defined as the occurrence of the final menstrual period between the ages of 40 and 45 [28]. FSD was defined as a total score on the FSFI questionnaire of less than 26.55 [20].

### 2.4. Statistical Analysis

Categorical data were presented as numbers and percentages, while continuous data were reported as means with standard deviations (SD) or medians with interquartile ranges (IQR), as appropriate. The Student’s *t*-test and Mann–Whitney U test were employed to assess differences in continuous variables between patients with and without FSD, as applicable. Associated factors that affected sexual function were analyzed and reported as an odds ratio (OR) with its 95% confidence interval (95%CI). Multivariable logistic regression was applied to investigate the factors associated with FSD among SSc patients. The *p* value < 0.05 was statistically significant. Data analysis was performed by the STATA version 16.0 (StataCorp., College Station, TX, USA).

## 3. Results

### 3.1. Patient Characteristics

A total of 31 eligible participants met the inclusion criteria. However, six patients were excluded from the analysis due to incomplete responses on the Thai FSFI questionnaire. Consequently, 27 participants were included in the final analysis, with a mean age of 39.4 ± 5.2 years (range: 22–45 years). Eleven participants had comorbid conditions, including atherosclerotic risk (8 cases; 29.6%), primary hyperthyroidism (2 cases; 7.4%), and primary hypothyroidism (1 case; 3.7%). None had a previous history of coronary artery disease. The average FSFI score was 23.7 ± 6.6. Fourteen participants were identified as having FSD, with a prevalence of 51.9% (95% CI: 31.9–71.3). The majority (66.7%) belonged to the dcSSc subset, with a mean disease duration of 9.9 ± 7.9 years. Raynaud’s phenomenon, ILD, and salt-and-pepper skin appearance were the most common characteristics among participants (62.9%, 55.6%, and 51.9%, respectively). The demographic data are summarized in Table 1.

### 3.2. Obstetrics and Gynecologic History

A total of 18 out of the 27 participants had been pregnant at least once. Eleven participants (40.7%) were postmenopausal, and three women (11.1%) reported vaginal dryness. The most common methods of contraception were tubal sterilization (30.8%) and condom use (30.8%) (Table 1).

### 3.3. Clinical Association with Sexual Dysfunction

When comparing the clinical characteristics of SSc patients with and without FSD, the age at disease onset was higher in patients with FSD compared to those without FSD (32.6 vs. 26.6 years), although this difference was not statistically significant (*p* = 0.06). Similarly, the duration of disease was longer in patients with FSD than in those without FSD (12.1 vs. 7.5 years, *p* = 0.14). Patients with FSD tended to have more frequent telangiectasia, both at disease onset and at the time of the study, but less frequent gastrointestinal involvement (esophageal, stomach, and intestinal involvement) at onset compared to those without FSD. No significant differences were found in other SSc characteristics or in obstetric and gynecologic history between the FSD and non-FSD groups. Regarding medical treatments, the use of cyclophosphamide (CYC), including treatment duration and age at initiation, was slightly higher in the FSD group compared to the non-FSD group, though this difference was not statistically significant. A similar trend was observed for prednisolone use. The clinical associations between the FSD and non-FSD groups, based on univariable analysis, are summarized in Table 2.

### 3.4. FSFI Score Domain

Patients with FSD had significantly lower scores in sexual arousal, lubrication, orgasm, sexual satisfaction, and total sexual function compared to those without FSD (*p* < 0.01 for all). Although sexual desire and pain scores were also lower in the FSD group, these differences did not reach statistical significance (*p* = 0.09 and *p* = 0.06, respectively) (Table 3).

## 4. Discussion

Sexual dysfunction is one of the most common issues among couples in the general population. Our study employed a cross-sectional design to determine the prevalence of FSD among women of childbearing age with SSc, using the FSFI questionnaire to assess sexual function. The questionnaire evaluated sexual feelings and responses over the past 4 weeks and has demonstrated a validity of 0.99 in the Asian population [29]. We also investigated whether SSc clinical characteristics, treatments affecting ovarian function, and obstetric and gynecologic histories were associated with FSD.

More than half of the SSc patients (51.9%) were identified as having FSD in our study. This FSD rate was slightly higher than that reported in the study by Alidost et al. in 2021 [30]. The authors conducted a systematic review that included women of reproductive age (15–49 years) without specifying comorbid diseases, analyzing data from 21 studies across various countries (China, Colombia, Egypt, Iran, India, Korea, Malaysia, Nigeria, and Turkey). The pooled prevalence of FSD in the study was 50.8% (95% CI: 41.73–59.78); however, there was significant heterogeneity between the studies (I^2^ = 99%) [30]. Compared to the previous study by Levis et al., which analyzed FSD in UK SSc patients, the FSD rate in our SSc patients was lower (51.9% vs. 61%) [11]. Another study by the same authors in 2012 found an FSD rate of 61.8% among sexually active SSc patients [31]. The systematic review reported that the prevalence of FSD among SSc patients varied from 46% to 86%, based on eight studies that included female SSc patients of all ages [32]. The difference in the FSD rate in our study compared to previous studies may be explained by differences in the populations included. Our study focused on individuals aged 18–45 years, while both studies by Levis et al. included all sexually active patients, regardless of age. Additionally, differences in race and culture may also impact sexual interest and function [33]. Although there are differences in the case populations, sexual dysfunction is a common problem among female SSc patients.

Older age was associated with a higher rate of sexual dysfunction. We observed that older age at onset tended to correlate with FSD, a finding that was not unexpected. This is because older populations are more prone to sexual dysfunction and reduced sexual activity as a result of hormonal and physical changes. Early menopause increases the likelihood of sexual dysfunction in women. Postmenopausal women have a high incidence of FSD, with rates exceeding 80% [34]. Multifactorial factors affecting sexual function include health, physical condition, and social environment. These factors may explain the higher FSD rates reported in previous studies that included SSc patients older than those in our study.

A high prevalence of sexual dysfunction has been reported in women with underlying diseases [35,36,37]. In systemic autoimmune diseases, women with SSc or Sjogren’s syndrome have the highest level of sexual dysfunction [38]. In addition, we found that the FSD rate was higher in women with dcSSc than in those with lcSSc (71.4% vs. 28.6%), although this difference was not statistically significant. The FSD rate among our dcSSc patients was slightly higher than in the previous study by Levis et al., which reported an FSD rate of around 68% in dcSSc, while the FSD rate in lcSSc was lower than in their study, which was around 62% [31]. The findings indicate that FSD is common in dcSSc. The pathophysiological changes in SSc, regardless of the subset, impact sexual function due to limitations in movement, pain, and psychological perception [39,40]. These factors may lead to sexual inactivity and/or sexual dysfunction in SSc patients.

Our study found that the presence of telangiectasia is more likely to be associated with FSD. Physiological findings, such as telangiectasia and facial skin involvement, were linked to greater dissatisfaction, increased social discomfort, and body image distress [41]. However, the exact explanation is uncertain. The result might be due to random chance or be explained by psychologic problems that might induce mental stress and sexual function impairment [39].

We hypothesized that longer duration and higher cumulative doses of CYC may be associated with FSD, compared to those who have never taken CYC or have used it for a shorter duration with a lower cumulative dose. Based on our findings, patients with FSD had a higher rate of CYC use and longer treatment duration than those without FSD, although the cumulative dose was lower. Jutiviboonsuk et al. reported that SSc patients who received CYC were more likely to experience premature ovarian insufficiency and early menopause [42]. This pathophysiologic factor is proposed to be the explanation for the sexual function decline in SSc women who were treated with CYC. However, we did not find these effects in our study due to a low power of analysis related to the small sample size. In addition, the participants in our study were in a younger age group, which was an important factor in sexual desire.

From our observation, SSc women with FSD scored lower in the arousal, lubrication, orgasm, and satisfaction domains compared to women without FSD. According to the sexual response cycle during sexual activity, the sexual response consists of three phases: desire, arousal, and orgasm. These phases depend on emotional responses and physiologic adaptations [43]. Physiological changes initially occur during the arousal phase, particularly an increase in genital blood flow, which leads to the formation of a neurogenic transudate that lubricates the vagina. Therefore, a decrease in or absence of the arousal phase results in reduced lubrication, leading to genital pain and dissatisfaction during sexual activity [44]. According to physiological changes in SSc, collagen change or a lack of smooth muscle relaxation in the vagina of women with SSc may impact sexual function [45]. In addition, the majority of SSc women typically receive CYC for immunosuppression. However, CYC not only affects fibrotic cells but also impacts gonadal cells [42]. The gonadotoxic effect of CYC induces early menopause by decreasing estrogen, progesterone, and androgen production in the ovaries. The reduction in these hormones leads to FSD by lowering sexual desire, decreasing clitoral and vaginal blood flow, causing a lack of vaginal lubrication, reducing genital sensation, and resulting in atrophy of the vaginal mucosa and smooth muscle [45,46]. A decrease in genital sensation leads to a reduced frequency of orgasm in these patients.

When focusing on gynecological symptoms, vaginal dryness was not common in our study despite the presence of FSD. These findings differ from those of Impens et al. [13], who investigated sexual function and activity in 78 female SSc patients of all ages. The authors reported vaginal dryness in 41.7% of participants, while our study found vaginal dryness in only 11.1% [13]. These differences could be explained by variations in the study populations. Our population was limited to women of reproductive age (18–45 years) who were sexually active, so vaginal dryness may not have been a prominent issue in this group. Due to the specific selection of a reproductive-age group, there were a small number of eligible participants, as the prevalence of SSc is not predominantly in this age group but peaks in those aged 60–69 years [4]. The results indicated that vaginal dryness may not link to FSD in these age groups.

Some contraceptives, particularly oral pills, have been reported to affect female sexual function. This may be due to mood changes or negative effects on body image, such as weight gain [47]. Our study found that the use of either hormonal or non-hormonal contraception did not affect sexual function among SSc patients. This finding was consistent with the previous report by Burrows et al., who described that a small percentage of women experienced decreased libido when using hormonal contraception, while the majority were unaffected, and some even reported an increase in libido [48]. However, since only a small number of our patients used oral pills, we cannot make definitive conclusions about their impact on sexual function in SSc. Further studies focusing on the effects of hormonal treatment on sexual dysfunction in SSc are recommended.

Although the FSFI score is a simple and inexpensive tool for evaluating sexual function, it is a subjective measurement and may result in a recall bias and non-response bias in cases of hesitancy in expressing responses to sensitive questions. We suggest using objective tools such as clitoral temperature, vaginal pH, vaginal Doppler ultrasound, vaginometry, genital biothesiometry, etc., to assess the physiological change in sexual function for further study. Despite the potential precision of objective measurements, these measurements have not been shown to be a reliable indicator of subjective arousal in women. Research has shown that there is a poor correlation between objective and subjective methods [49]. The fact that subjective assessments are affordable, standardized, simple to administer and score, and have normative values gives them a substantial advantage over objective techniques [50].

Both men and women with SSc also have sexual problems. In men, erectile dysfunction is common, with a reported prevalence of around 80–88% [51,52,53,54]. The FSD rate in women, including our study, was around 50–62% [11,31]. The causes and pathogenesis of both erectile dysfunction in men and sexual dysfunction in women with SSc remain unclear. Potential etiologic factors for erectile dysfunction in men include older age, medications, and vasculogenic, psychogenic, and neurogenic causes [55,56,57,58]. However, FSD in SSc is not well understood. Although sexual dysfunction may not impact disease prognosis as significantly as internal organ involvement in SSc, FSD contributes to a lower quality of life [11,13,31] and may cause a family problem in both sexes.

Our study had some limitations: (a) a small sample size, which reduced the power of the analysis. According to the epidemiology of SSc in our country, the peak prevalence and incidence occur among individuals aged 60–69 years. Only a minority of cases are younger than 40 years [4], which corresponds to the reproductive age and was the target population of our study. The number of participants in our study reflects the overall low prevalence of SSc among women of reproductive age. We focused on individuals in their reproductive years, a group that is in the process of building families. Therefore, the results of this study are likely to contribute to counseling and family planning for patients in the future; (b) a single-center design, which means the findings may not be generalizable; (c) no control group for comparison because we aimed to evaluate the prevalence of FSD and associated factors focusing exclusively on female SSc patients. Therefore, we collected data only from SSc patients and we cannot conclude whether the prevalence of FSD among Thai SSc patients is higher than that of the general population; (d) the potential for non-response bias. In Thai culture, some individuals avoid discussing or expressing issues related to sexual topics, which may have made them hesitant to provide responses to the questionnaire; and (e) difficulty controlling confounders. The study may be affected by confounding variables such as psychological stress, cultural issues, and family relationships that are difficult to control, which could influence the findings related to FSD in SSc patients. Future studies with larger, more diverse samples and methods to mitigate cultural and psychological barriers, including established scales for the evaluation of neuropsychological status, are needed for a more comprehensive understanding.

However, the strengths of our study include the inclusion of SSc clinical characteristics at onset, at the time of the study, serology, potential medications that may affect sexual organ function, comorbid diseases, and obstetric and gynecologic history. The findings may provide valuable insights for improving care and planning family counseling for SSc patients.

## 5. Conclusions

FSD is a common problem among Thai female SSc patients of reproductive age. Older age at onset, disease severity, and long-term CYC use might be risk factors for impaired sexual function in SSc. Further research with a larger sample and objective tools for evaluating sexual function is needed to support these findings.

## Figures and Tables

**Table 1:** Demographic data.

Data	*N* = 27
Age at onset (years); mean ± SD	29.5 ± 8.5
Age on study date (years); mean ± SD	39.4 ± 5.6
BMI (kg/m^2^) mean ± SD	20.4 ± 3.5
Disease duration (years; mean ± SD	9.9 ± 7.9
Diffuse cutaneous SSc; n (%)	18 (66.7)
Anti-topoisomerase I positive; n (%)	16 of 24 (66.7)
Anti-centromere positive; n (%)	1 of 23 (4.3)
Comorbid diseases; n (%)	
Atherosclerotic risk; n (%)	8 (29.6)
Diabetes mellitus; n	0
Hypertension	0
Dyslipidemia	3
Overweight	4
Dyslipidemia and overweight	1
Primary hyperthyroidism; n (%)	2 (7.4)
Primary hypothyroidism; n (%)	1 (3.7)
Female sexual function	
Female Sexual Function Index score; mean ± SD	23.7 (6.6)
Sexual dysfunction; n (%)	14 (51.9)
SSc clinical characteristics at onset	
Raynaud’s phenomenon; n (%)	25 (92.6)
Salt-and-pepper skin; n (%)	19 (70.4)
Telangiectasia; n (%)	7 (25.9)
Hand deformity; n (%)	7 (25.9)
Synovitis; n (%)	6 (22.2)
Edematous skin; n (%)	11 (40.7)
Esophageal involvement; n (%)	13 (48.2)
Stomach involvement; n (%)	3 (11.1)
Intestinal involvement; n (%)	3 (11.1)
Interstitial lung disease; n (%)	11 (40.7)
Pulmonary arterial hypertension; n (%)	1 (7.1)
Pericardial effusion; n (%)	2 (7.4)
Left ventricular systolic dysfunction; n (%)	0
Diastolic dysfunction; n (%)	0
Renal crisis; n (%)	0
Modified Rodnan skin score; median (IQR)	10 (2–14)
Obstetrics and gynecologic history	
Parity	
None	9 (33.3)
≥1	18 (66.7)
Dysmenorrhea	11 (40.7)
Abnormal uterine bleeding	2 (7.4)
Early menopause	11 (40.7)
Vaginal dryness	3 (11.1)
Dyspareunia	4 (14.8)
Contraception use	13 (48.1)
Tubal sterilization	4 (30.8)
Condom	4 (30.8)
Oral combine pills	3 (23.1)
Medroxyprogesterone acetate injection	2 (15.4)
SSc characteristics on study date	
Raynaud’s phenomenon; n (%)	17 (62.9)
Salt-and-pepper skin; n (%)	14 (51.9)
Telangiectasia; n (%)	12 (44.4)
Hand deformity; n (%)	12 (44.4)
Synovitis; n (%)	1 (3.7)
Edematous skin; n (%)	0
Esophageal involvement; n (%)	8 (29.6)
Stomach involvement; n (%)	4 (14.8)
Intestinal involvement; n (%)	3 (11.1)
Interstitial lung disease; n (%)	15 (55.6)
Pulmonary arterial hypertension; n (%)	1 (3.7)
Modified Rodnan skin score; median (IQR)	2 (0–6)

BMI: body mass index; SSc: systemic sclerosis; SD: standard deviation; IQR: interquartile range; mRSS: modified Rodnan skin score.

**Table 2:** Univariable analysis of clinical association with FSD.

Characteristics	FSD *N* = 14	No FSD *N* = 13	OR (95% CI)	*p*-Value
Age at onset (years); mean ± SD	32.6 ± 6.6	26.6 ± 8.5	-	0.06
Age on study date (years); mean ± SD	38.7 ± 6.4	40.1 ± 4.9	-	0.54
BMI (kg/m)^2^; mean ± SD	20.7 ± 3.8	20.0 ± 3.2	-	0.58
Disease duration (years); mean ± SD	12.1 ± 9.5	7.5 ± 5.3	-	0.14
Limited cutaneous SSc; n (%)	4 (28.6)	5 (38.6)	1	
Diffuse cutaneous SSc; n (%)	10 (71.4)	8 (61.5)	1.56 (0.24–10.68)	0.59
Anti-topoisomerase I positive; n (%)	7 (58.3)	9 (75.0)	0.47 (0.05–3.54)	0.39
Comorbid diseases				
Cardiovascular diseases; n (%)	5 (35.7)	3 (23.1)	1.85 (0.26–15.2)	0.52
Primary hyperthyroidism; n (%)	1 (7.1)	1 (7.7)	0.92 (0.01–78.42)	0.96
Primary hypothyroidism; n (%)	0 (0)	1 (7.7)	NA	NA
SSc clinical characteristics at onset				
Raynaud’s phenomenon; n (%)	12 (85.7)	13 (100)	NA	NA
Salt-and-pepper skin; n (%)	10 (71.4)	9 (69.2)	1.11 (0.15–7.96)	0.90
Telangiectasia; n (%)	6 (42.9)	1 (7.7)	9.00 (0.78–447.59)	0.03
Hand deformity; n (%)	5 (35.7)	2 (15.4)	3.06 (0.37–37.84)	0.22
Synovitis; n (%)	3 (21.4)	3 (23.1)	0.91 (0.10–8.48)	0.92
Edematous skin; n (%)	5 (35.7)	6 (46.2)	0.65 (0.11–3.90)	0.58
Esophageal involvement; n (%)	5 (35.7)	8 (61.5)	0.35 (0.05–2.10)	0.18
Stomach involvement; n (%)	1 (7.1)	2 (15.4)	0.42 (0.01–9.46)	0.50
Intestinal involvement; n (%)	0	3 (23.1)	NA	NA
Interstitial lung disease; n (%)	7 (50.0)	4 (30.8)	2.25 (0.37–14.81)	0.31
Pulmonary arterial hypertension; n (%)	1 (7.1)	0	NA	NA
Modified Rodnan skin score; median (IQR)	11 (2–14)	10 (3–15)	-	0.76
Obstetrics and gynecologic history				
Parity				
None	4 (28.6)	5 (38.3)	0.59 (0.31–7.81)	0.60
≥1	10 (71.4)	8 (61.5)	1	
Abnormal uterine bleeding	1 (7.1)	1 (7.7)	0.92 (0.01–78.42)	0.96
Early menopause	7 (50.0)	4 (30.8)	2.25 (0.37–14.80)	0.31
Vaginal dryness	1 (7.1)	3 (23.1)	0.26 (0.005–3.95)	0.24
Dyspareunia	2 (14.3)	2 (15.4)	0.92 (0.11–7.67)	0.94
Contraception use	6 (42.9)	7 (53.9)	0.64 (0.11–3.73)	0.57
Tubal sterilization	1 (16.7)	3 (42.9)		
Condom	2 (33.3)	2 (28.6)		
Oral combine pills	1 (16.7)	2 (28.6)		
Medroxyprogesterone acetate	2 (33.3)	0		
SSc characteristics on the study date				
Raynaud’s phenomenon; n (%)	8 (57.1)	9 (69.2)	0.59 (0.09–3.70)	0.52
Salt-and-pepper skin; n (%)	8 (57.1)	6 (46.1)	1.56 (0.27–9.19)	0.57
Telangiectasia; n (%)	9 (64.3)	3 (23.1)	6.00 (0.87–47.52)	0.03 *
Hand deformity; n (%)	7 (50.0)	5 (38.5)	1.60 (0.27–9.68)	0.55
Synovitis; n (%)	0	1 (7.7)	NA	NA
Edematous skin; n (%)	0	0	NA	NA
Esophageal involvement; n (%)	5 (35.7)	3 (23.1)	1.85 (0.26–15.15)	0.47
Stomach involvement; n (%)	3 (21.4)	1 (7.7)	3.27 (0.21–185.84)	0.32
Intestinal involvement; n (%)	1 (7.1)	2 (15.4)	0.42 (0.01–9.46)	0.50
Interstitial lung disease; n (%)	8 (57.1)	7 (53.9)	1.14 (0.19–6.71)	0.86
Pulmonary arterial hypertension; n (%)	1 (7.1)	0	NA	NA
Modified Rodnan skin score; median (IQR)	1 (0–6)	2 (0–2)	-	0.98
Medications				
Cyclophosphamide				
Ever used; n (%)	8 (57.1)	6 (46.2)	1.55 (0.34–7.11)	0.57
Cumulative dose (g); median (IQR)	15.0 (7.9–29.4)	18.0 (13.6–19.2)	-	0.64
Duration of used (days); mean ± SD	18.5 ± 11.0	15.2 ± 7.5	-	0.51
Starting age (years); mean ± SD	34.8 ± 5.8	33.3 ± 7.4	-	0.67
Prednisolone				
Ever used; n	12 (85.7)	10 (76.9)	1.80 (0.17–25.07)	0.56
Cumulative dose (g); median (IQR)	7.1 (1.1–18.0)	5.4 (2.7–9.1)	-	0.97
Duration of used (days); mean ± SD	37.2 ± 34.9	35.4 ± 22.7	-	0.89
Starting age (years); mean ± SD	32.5 ± 8.1	33.3 ± 6.3	-	0.79

* Statistically significant. FSD: female sexual dysfunction; OR: odds ratio; BMI: body mass index; SSc: systemic sclerosis; SD: standard deviation; IQR: interquartile range; mRSS: modified Rodnan skin score; NA: no data available due to statistical limitation.

**Table 3:** Female Sexual Function Index score in SSc women with and without FSD.

Domain	FSD (*N* = 14)	No FSD (*N* = 13)	Mean Difference (95% CI)	*p*-Value
Sexual desire; mean (SD)	5.3 (1.3)	4.6 (0.6)	−0.69 (−1.51, −0.11)	0.09
Sexual arousal; mean (SD)	2.6 (1.4)	5.2 (0.4)	2.62 (1.78, 3.47)	<0.001 *
Lubrication; mean (SD)	2.2 (1.6)	5.3 (0.7)	3.15 (2.13, 4.17)	<0.001 *
Orgasm; mean (SD)	2.4 (1.7)	4.9 (0.8)	2.52 (1.45, 3.60)	<0.001 *
Pain; mean (SD)	2.8 (1.4)	3.7 (0.9)	0.89 (−0.04, 1.82)	0.06
Sexual satisfaction; mean (SD)	3.2 (2.5)	5.7 (1.0)	2.48 (0.94, 4.02)	0.003 *
Total score; mean (SD)	18.5 (4.4)	29.4 (2.2)	10.97 (8.18, 13.76)	<0.001 *

* Statistically significant. FSD: female sexual dysfunction; SD: standard deviation.

## Data Availability

The datasets used and/or analyzed during the current study are available from the corresponding author upon reasonable request.
